# The Effect of Heat Treatment after Hydrothermal Reaction on the Lithium Storage Performance of a MoS_2_/Carbon Cloth Composite

**DOI:** 10.3390/ma16247678

**Published:** 2023-12-17

**Authors:** Xintong Li, Chonggui Li, Qi Yang

**Affiliations:** School of Materials Science and Engineering, Shanghai University of Engineering Science, Shanghai 201620, China

**Keywords:** molybdenum disulfide, carbon cloth, heat treatment, anode material, lithium-ion batteries

## Abstract

In this study, 1T phase MoS_2_ nanosheets were synthesized on the surface of a carbon cloth via a hydrothermal reaction. After heat treatment, the 1T phase MoS_2_ was transformed into the 2H phase with a better capacity retention performance. As an anode material for lithium-ion batteries, 2H phase MoS_2_ on the carbon cloth surface delivers a capacity of 1075 mAh g^−1^ at a current density of 0.1 A g^−1^ after 50 cycles; while the capacity of the 1T phase MoS_2_ on the surface of the carbon cloth without heat treatment fades to 528 mAh g^−1^. The good conductivity of a carbon cloth substrate and the separated MoS_2_ nanosheets help to increase the capacity of MoS_2_ and decrease its charge transfer resistance and promote the diffusion of lithium ions in the electrode.

## 1. Introduction

Since the invention of lithium-ion batteries (LIBs) in 1991, they have become the primary power source for portable electronic devices due to their high-energy density, good cycling stability, and long service life [1,2]. In recent years, due to the development of electric vehicles, material scientists have been required to develop LIBs with higher energy density [3,4,5]. However, the currently commercialized graphite anode material cannot meet the increasing energy density requirements of LIBs because of its low theoretical capacity (372 mAh g^−1^) [6]. Transition metal sulfides with a higher theoretical capacity than the graphite anode material have attracted considerable attention [7,8,9]. As one of transition metal sulfides, MoS_2_ has a theoretical specific capacity (670 mAh g^−1^), which is 1.8 times the theoretical capacity of graphite [10,11,12]. MoS_2_ has a layered structure. Single-layer MoS_2_ consists of two layers of sulfur atoms sandwiched by a layer of molybdenum atoms [13]. Multilayer MoS_2_ is composed of several single-layer MoS_2_ connections, with a spacing of ~0.65 nm between layers. The layered structure of MoS_2_ facilitates the diffusion of lithium ions in the electrode and buffers the volume change during charge–discharge, which leads to its good cyclic reversibility [14,15]. However, as an inorganic material, MoS_2_ has poor conductivity. Ammonium molybdate, which is commonly used as a raw material for the hydrothermal synthesis of MoS_2_, is readily soluble in water but not in organic solvents. Due to the strong polarity of water, MoS_2_ synthesized with the hydrothermal method is prone to agglomerate. Large-sized aggregates will decrease the sites of electrochemical reactions and increase the diffusion distance of lithium ions in the electrode, resulting in a reduction in their electrochemical performance. Therefore, determining how to improve the conductivity of MoS_2_ and how to synthesize its nanostructures is an important strategy to enhance its electrochemical performance.

Carbonaceous materials have good conductivity, and their volume effect during charge–discharge is small [16,17]. Thus, as substrates, carbonaceous materials can improve the conductivity of transition metal sulfides and buffer their volume changes. A carbon cloth is a flexible carbonaceous material woven with carbon fibers [18]. Its good conductivity and mechanical flexibility make it an appropriate collector for flexible LIB electrodes.

Recently, there are some studies on the synthesis and lithium storage performance of MoS_2_/carbon cloth composites. Some effective strategies, such as the preparation of ultra-thin MoS_2_ nanosheets, the fabrication of 3D graphene/MoS_2_ spherical heterostructure [19], the N doping of MoS_2_ [20], the preparation of CdS@MoS_2_ core-shell structured nanospheres [21], and the P doping of MoS_2_ [22] have been conducted to improve the lithium storage performance of MoS_2_/carbon cloth composites. However, the effect of heat treatment after the hydrothermal reaction on their lithium storage performance has not been the subject of investigation in this study. Heat treatment will transform 1T phase MoS_2_ with a larger volume effect into 2H phase MoS_2_ with a smaller volume effect, which is beneficial for improving its capacity retention performance. Herein, MoS_2_ nanosheets were grown on the surface of the carbon cloth using a hydrothermal reaction and subsequent heat treatment was conducted to increase their electrochemical performance.

## 2. Experimental Procedure

### 2.1. Material Synthesis

Carbon cloth (WOS1011, CeTech Co., Ltd., Taiwan, China) was pretreated with 1M nitric acid (Shanghai Titan Scientific Co., Ltd., Shanghai, China) aqueous solution for 3 h, washed several times with deionized water, and then dried at 60 °C for 12 h. Ammonium molybdate tetrahydrate (0.3 g) (Shanghai Titan Scientific Co., Ltd., Shanghai, China) and thiourea (Shanghai Titan Scientific Co., Ltd., Shanghai, China) with the S/Mo molar ratios of 4, 5, and 6 were weighed and then dissolved in 35 mL deionized water under magnetic stirring for 30 min to prepare the solution. The obtained solution and 4 pieces of pretreated carbon cloth (10 mm × 10 mm) were transferred to a Teflon-lined, stainless-steel autoclave (50 mL) and kept at 180 °C for 6 h. After being cooled to room temperature, the carbon cloth with MoS_2_ nanosheets was washed several times with deionized water. Subsequently, the washed carbon cloth was dried at 60 °C for 12 h. The MoS_2_/carbon cloth composite synthesized with hydrothermal reaction was denoted as MoS_2_/CC-No HT. The MoS_2_/CC-No HT was placed in the tube furnace under Ar atmosphere, then sintered at 500 °C and maintained for 2 h with a heating rate of 3 °C min^−1^. The obtained MoS_2_/carbon cloth composite was denoted as MoS_2_/CC-HT. For comparison, MoS_2_ powder was synthesized without the addition of carbon cloth. The MoS_2_ powder synthesized with hydrothermal reaction was denoted as MoS_2_-No HT. The MoS_2_ powder synthesized with hydrothermal reaction and subsequent heat treatment was denoted as MoS_2_-HT.

### 2.2. Material Characterizations

X-ray diffractometer (XRD, Panalytical X’Pert3 Powder, PANalytical B.V., Almelo, The Netherlands) was utilized to measure crystal structure. X-ray photoelectron spectroscopy (XPS, Thermo Scientific K-Alpha, Thermo Fisher Scientific, Waltham, MA, USA) was employed to analyze the chemical state and elemental valence of the samples. Scanning electron microscope (SEM, Hitachi S4800, Hitachi, Tokyo, Japan) and transmission electron microscope (TEM, JEOL JEM-F200, JEOL, Tokyo, Japan) were investigated and used to conduct the morphological observations and microstructural analysis of the samples. An ASAP2460 (Micromeritics, Atlanta, GA, USA) instrument was used to test the specific surface area and pore structure.

### 2.3. Electrochemical Measurements

The electrochemical performance of the composites was assessed utilizing CR2032-type half coin cells. The coin cells were assembled in a glove box with both H_2_O and O_2_ contents below 0.1 ppm. MoS_2_/CC-HT and MoS_2_/CC-No HT were utilized as working electrodes without any intervening steps. The weight of the active substances loaded on the carbon cloth was calculated to be approximately 2.3 ± 0.2 mg cm^−2^. For MoS_2_-HT and MoS_2_-No HT powders, it is necessary to fabricate working electrodes before the assembly of coin cells. The powder samples were prepared as a uniform and stable slurry by mixing the active materials, carbon black, and polyvinylidene fluoride (PVDF) in N-methyl-2-pyrrolidone (NMP) solvent with a weight ratio of 8:1:1. The slurry was applied to the surface of copper foil and dried at 60 °C for 12 h in a vacuum oven. Lithium foil was used as the counter electrode, while the Celgard 2400 membrane functioned as the separator. The electrolyte was 1.0 M LiPF_6_ in a mixed solution that blended ethylene carbonate (EC), diethyl carbonate (DEC), and methyl ethyl carbonate (EMC) at a volume ratio of 1:1:1. The cyclic voltammetry (CV) and electrochemical impedance spectroscopy (EIS) measurements were performed on the CHI660E (Shanghai Chenhua Instrument Co., Ltd., Shanghai, China) electrochemical workstation. The galvanostatic charge/discharge test was investigated with a Neware-CT3008 instrument (NEWARE Technology Limited, Shenzhen, China).

## 3. Results and Discussion

**Figure 1:**
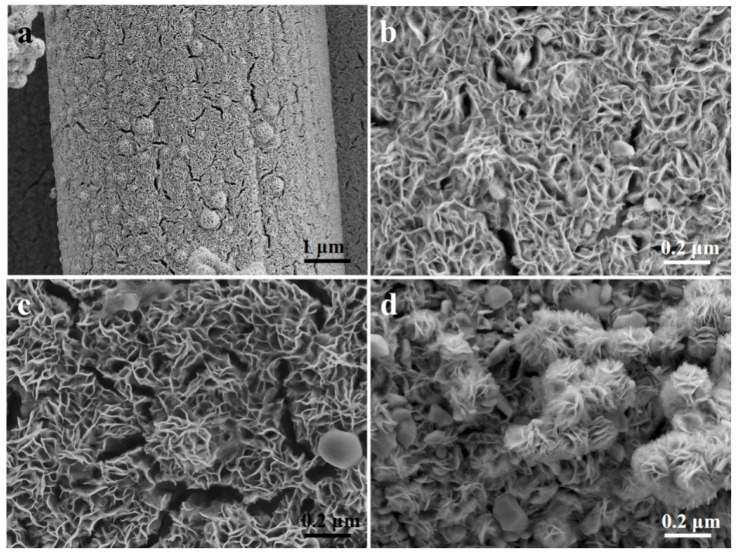
SEM images of MoS_2_/CC-HT synthesized under the different S/Mo molar ratios of: (**a**,**b**) 5; (**c**) 6; (**d**) 4.

**Figure 2:**
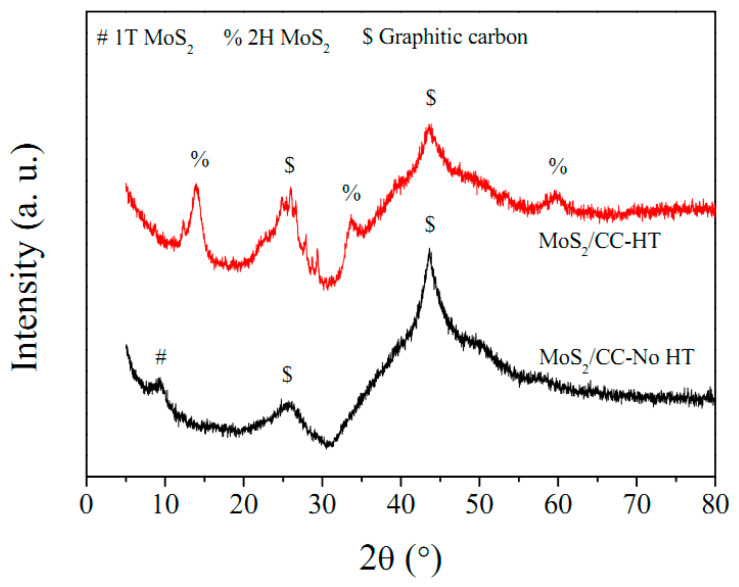
XRD patterns of MoS_2_/CC-HT and MoS_2_/CC-No HT.

Figure 1 shows the SEM images of MoS_2_/CC-HT synthesized under different S/Mo molar ratios. The S/Mo molar ratio has a significant effect on the morphology of MoS_2_/CC-HT. As exhibited in Figure 1a,b, it can be seen that the MoS_2_ in MoS_2_/CC-HT synthesized under the S/Mo molar ratio of 5 is ultra-thin nanosheets. In Figure 1c, the MoS_2_ in MoS_2_/CC-HT synthesized under the S/Mo molar ratio of 6 shows a similar morphology to the MoS_2_ in MoS_2_/CC-HT synthesized under the S/Mo molar ratio of 5. From Figure 1d, it can be observed that the MoS_2_ in MoS_2_/CC-HT synthesized under the S/Mo molar ratio of 4 is composed of particles with the size of 50–200 nm and flower-like nanostructure assembled by nanosheets. Ultra-thin nanosheets will provide many electrochemical reaction sites, decrease the diffusion distance of lithium ions, and reduce the absolute volume change caused by lithiation–delithiation due to their large specific surface area and ultra-thin thickness. Therefore, in the following results and discussion, MoS_2_/CC-HT and MoS_2_/CC-No HT were synthesized under the S/Mo molar ratio of 5. For comparison, MoS_2_-HT and MoS_2_-No HT were also synthesized under the S/Mo molar ratio of 5. MoS_2_/CC-HT and MoS_2_/CC-No HT have the same morphology. Heat treatment at 500 °C only changes the phase of MoS_2_/CC-No HT (see the XRD analysis in Figure 2), not its morphology.

Figure 2 illustrates the XRD patterns for MoS_2_/CC-HT and MoS_2_/CC-No HT. Two peaks at 25.6 and 43.6° correspond to the (002) and (100) crystal planes of graphitic carbon, respectively. These characteristic XRD peaks are from the carbon cloth [23,24,25]. In the XRD pattern of MoS_2_/CC-No HT, the peak detected at 9.4° is assigned to the (002) crystal plane of 1T phase MoS_2_ [26,27]. The peak intensity is weak, indicating the low crystallinity of the MoS_2_. In the XRD pattern of MoS_2_/CC-HT, the peaks observed at 13.8, 33.6, and 59.5° are attributed to the (002), (100), and (110) crystal planes, respectively, of 2H phase MoS_2_ (JCPDS No. 37-1492). The increase in peak intensity indicates an increase in the crystallinity of the 2H phase MoS_2_. According to the Scherrer equation, the average grain size of the 2H phase MoS_2_ is 9.9 nm. The grain boundaries are rich in defects which provide channels for the diffusion of lithium ions. Thus, the ultrafine average grain size is conducive to the diffusion of lithium ions in the electrode.

**Figure 3:**
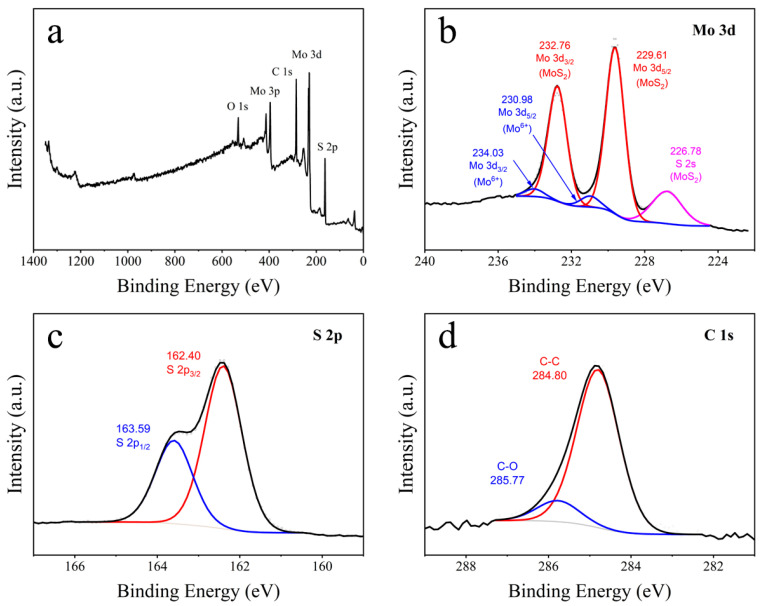
XPS spectra of MoS_2_/CC-HT: (**a**) overall spectrum; (**b**) Mo 3d; (**c**) S 2p; (**d**) C 1s.

X-ray photoelectron spectroscopy (XPS) is used to analyze the chemical states and surface elemental composition of MoS_2_/CC-HT. As shown in Figure 3a, the XPS survey spectrum of MoS_2_/CC-HT, the spectrum exhibits characteristic peaks of Mo 3d, S 2p, C 1s, and O 1s, indicating the existence of MoS_2_ and carbon in MoS_2_/CC-HT. The characteristic peaks of O 1s are attributed to the water absorbed on the surface of MoS_2_/CC-HT. The center, FWHM, and area percentage for the deconvoluted peaks of Mo 3d, S 2p, and C 1s are presented in Tabs. S1 to S3, respectively (Supplementary Materials). Figure 3b shows the XPS high-resolution spectrum of Mo 3d. The peaks at 232.76 and 229.61 eV are related to Mo 3d_3/2_ and Mo 3d_5/2_ of Mo^4+^ in MoS_2_, respectively [28,29]; the peaks at 234.03 and 230.98 eV assigned to Mo 3d_3/2_ and Mo 3d_5/2_ of Mo^6+^ are related to the Mo-O-C bond between carbon fibers and MoS_2_ nanosheets [23,30]; the peak at 226.78 eV corresponds to S 2s in MoS_2_. Figure 3c shows the high-resolution XPS spectrum of S 2p; the separated peaks at 163.59 and 162.40 eV correspond to S 2p_1/2_ and S 2p_3/2_ of S^2−^, respectively [31]. In Figure 3d, the XPS high-resolution spectrum of C 1s exhibited two separated peaks at 284.80 and 285.77 eV, which are assigned to C-C and C-O bonds, respectively [28,32].

**Figure 4:**
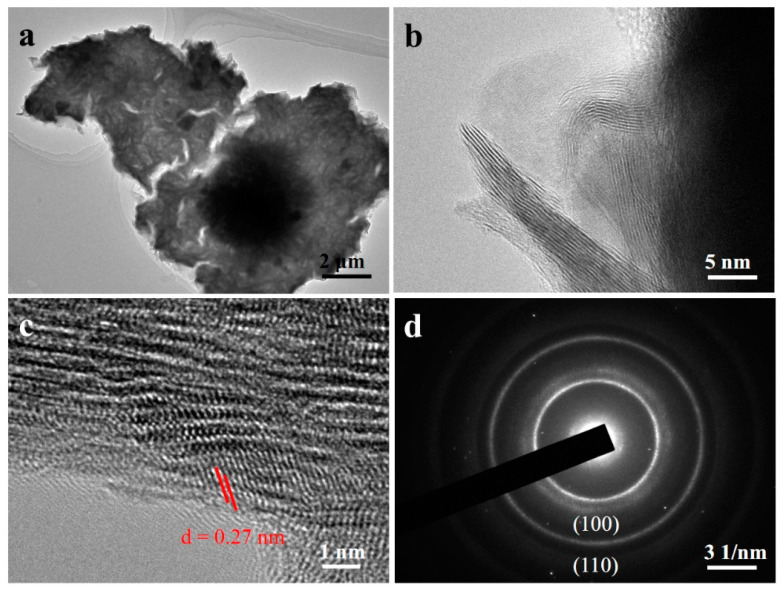
MoS_2_/CC-HT: (**a**,**b**) TEM images; (**c**) HRTEM image; (**d**) SAED pattern.

The TEM, HRTEM, and SAED analyses of MoS_2_ in MoS_2_/CC-HT are shown in Figure 4. The MoS_2_ in MoS_2_/CC-HT exhibits a sheet-like structure (Figure 4a), and the lattice fringes of MoS_2_ can be observed at its edges (Figure 4b). Figure 4c demonstrates the HRTEM image of MoS_2_. It can be seen that its lattice stripes are irregular. The crystal plane spacing of ~0.27 nm corresponds to the (100) crystal plane of 2H phase MoS_2_ (JCPDS No. 37-1492). The SAED image of MoS_2_ presented in Figure 4d illustrates its polycrystalline characteristics. The diffraction rings can be labeled as the (100) and (110) crystal planes of 2H phase MoS_2_ (JCPDS No. 37-1492).

**Figure 5:**
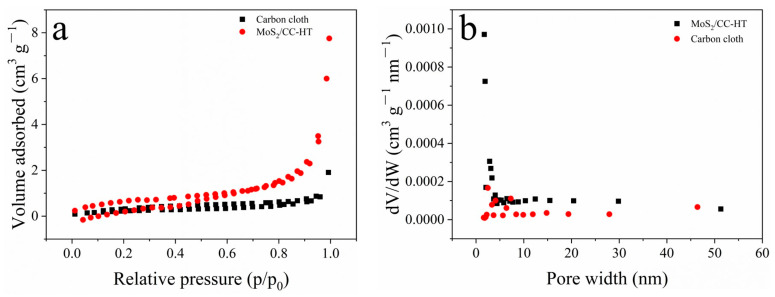
MoS_2_/CC-HT and carbon cloth: (**a**) N_2_ adsorption-desorption isotherms; (**b**) pore size distribution curves.

The specific surface area and pore structure analysis of MoS_2_/CC-HT and the carbon cloth are demonstrated in Figure 5. From Figure 5a, it can be seen that the N_2_ adsorption–desorption isotherms of MoS_2_/CC-HT and the carbon cloth belong to Type IV [33]. Calculated with the BET method, the specific surface areas of MoS_2_/CC-HT and the carbon cloth are 2.41 and 0.86 m^2^ g^−1^, respectively [33]. By removing the influence of the carbon cloth, it can be estimated that the specific surface area of MoS_2_ in MoS_2_/CC-HT is ~10.16 m^2^ g^−1^. From Figure 5b, it can be seen that MoS_2_/CC-HT has a rich micro-porous structure. The large specific surface area and rich micro-porous structure will increase electrochemical reaction sites and promote the lithium-ion diffusion in the electrode.

MoS_2_/CC-HT is composed of MoS_2_ and a carbon cloth. Thus, the electrochemical reactions related to its charge–discharge can be described by the following equations [23,28,32]:

(1) $$MoS_{2}+xLi^{+}+xe^{-}\to Li_{x}MoS_{2}$$

(2) $$Li_{x}MoS_{2}+\left(4-x\right)Li^{+}+\left(4-x\right)e^{-}\to Mo+2Li_{2}S$$

(3) $$2Li_{2}S-4e^{-}\to 2S+4Li^{+}$$

(4) $$Mo+2Li_{2}S-4e^{-}\to MoS_{2}+4Li^{+}$$

(5) $$Li+C+xe^{-}\leftarrow \to Li_{x}C$$

**Figure 6:**
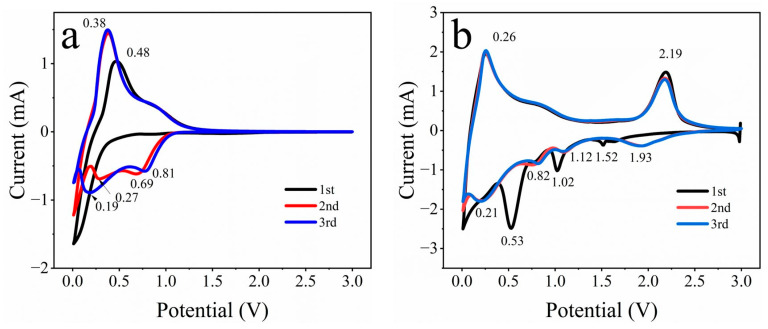
CV curves of: (**a**) carbon cloth; (**b**) MoS_2_/CC-HT.

Figure 6a demonstrates the CV curves of the carbon cloth. In the first scanning, the cathode peak around 0.01 V corresponds to Li^+^ insertion into the carbon cloth and the formation of solid electrolyte interface (SEI) film, and the anode peak at 0.48 V corresponds to Li^+^ de-insertion in the carbon cloth [34]. In the second scanning, the cathode peaks at 0.01, 0.27, and 0.69 V are related to Li^+^ insertion of the carbon cloth, and the anode peak at 0.38 V is related to Li^+^ de-insertion of the carbon cloth. In the third scanning, the cathode peaks at 0.01, 0.19 and 0.81 V can be assigned to Li^+^ insertion of the carbon cloth, and the anode peak at 0.38 V can be assigned to Li^+^ de-insertion of the carbon cloth. Figure 6b demonstrates the CV curves of MoS_2_/CC-HT. In the first cathode scanning, the two cathode peaks appear at 1.52 and 1.02 V, which are attributed to the insertion of Li^+^ into the MoS_2_ layer to form Li_x_MoS_2_ [35]; the cathode peak at 0.53 V is attributed to the formation of an SEI film, accompanied by the decomposition of Li_x_MoS_2_ to form metal Mo and Li_2_S [36], whereas the cathode peak at 0.01 V is attributed to the intercalation of Li^+^ in the carbon cloth. In the first anode scanning, the anode peak at 0.26 V is related to the de-intercalation Li^+^ in the carbon cloth, while the anode peak at 2.19 V is related to the oxidation process of LiS_2_ to S and Mo to MoS_2_ [37]. The CV curves of the second and third cycles almost coincide with each other, indicating that MoS_2_/CC-HT has good cyclic repeatability. In the second and third scanning, the cathode peak related to the formation of Li_x_MoS_2_ by the insertion of Li^+^ in MoS_2_ layers shifts to 1.93 V; the cathode peak related to the decomposition of Li_x_MoS_2_ to metallic Mo and Li_2_S shifts to 1.12 V [38]; the anode peaks at 0.01, 0.21, and 0.82 V can be assigned to the Li^+^ insertion of the carbon cloth.

**Figure 7:**
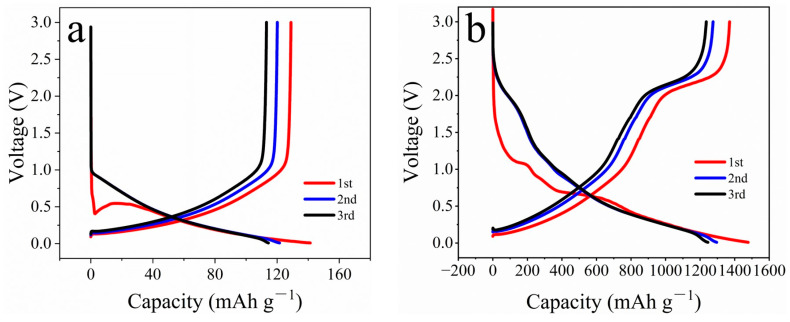
Charge–discharge profiles of: (**a**) carbon cloth; (**b**) MoS_2_/CC-HT.

Figure 7a demonstrates the charge–discharge curves of the carbon cloth. The discharge capacities of the carbon cloth are 141, 122, and 114 mAh g^−1^ in the first three cycles; its charge capacities are 129, 120, and 113 mAh g^−1^; and its Coulombic efficiencies are 91.5%, 98.4%, and 99.1%. In Figure 7b, the charge–discharge profiles of MoS_2_/CC-HT and the charge voltage plateau at 2.19 V corresponds to the oxidation of LiS_2_ to S and Mo to MoS_2_. The discharge capacities of MoS_2_/CC-HT are 1478, 1296, and 1245 mAh g^−1^ in the first three cycles; its charge capacities are 1370, 1274, and 1234 mAh g^−1^; and its Coulombic efficiencies are 92.7%, 98.3%, and 99.1%.

**Figure 8:**
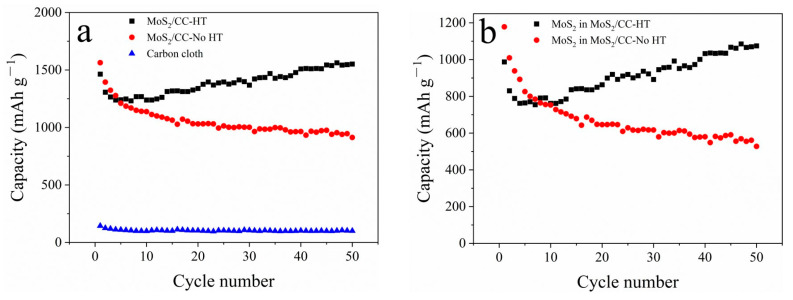
Cyclic performance: (**a**) MoS_2_/CC-HT, MoS_2_/CC-No HT, and carbon cloth; (**b**) MoS_2_ in MoS_2_/CC-HT and MoS_2_ in MoS_2_/CC-No HT.

The cyclic performance of MoS_2_/CC-HT, MoS_2_/CC-No HT, and the carbon cloth are shown in Figure 8a. The carbon cloth delivers a stable capacity of 110 mAh g^−1^ during cycling. The capacities of MoS_2_/CC-HT and MoS_2_/CC-No HT include the capacity of the carbon cloth and the capacity of MoS_2_ loaded on the surface of the carbon cloth. Therefore, the capacity of MoS_2_ in MoS_2_/CC-No HT and MoS_2_ in MoS_2_/CC-No HT (shown in Figure 8b) can be calculated by removing the capacity of the carbon cloth. The capacity of MoS_2_ in MoS_2_/CC-HT continuously increases to 1075 mAh g^−1^ at a current density of 0.1 A g^−1^ after 50 cycles, while the capacity of MoS_2_ in MoS_2_/CC-No HT continuously decreases to 528 mAh g^−1^. The MoS_2_ in MoS_2_/CC No HT is 1T phase, while the MoS_2_ in MoS_2_/CC HT is the 2H phase; 2H phase MoS_2_ has a better capacity retention performance than 1T phase MoS_2_. Therefore, with heat treatment, MoS_2_ on the surface of the carbon cloth is transformed from the 1T phase to the 2H phase, resulting in the better capacity retention performance of MoS_2_/CC-HT.

**Figure 9:**
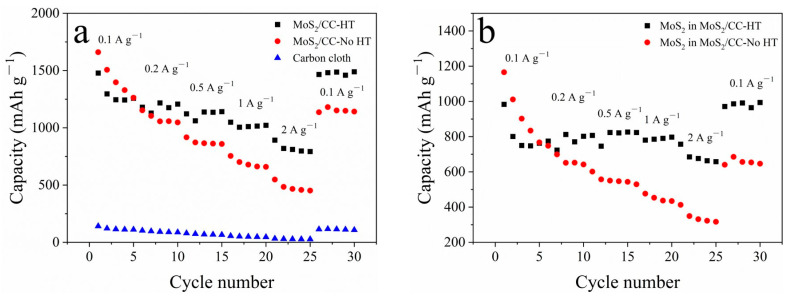
Rate performance: (**a**) MoS_2_/CC-HT, MoS_2_/CC-No HT, and carbon cloth; (**b**) MoS_2_ in MoS_2_/CC-HT and MoS_2_ in MoS_2_/CC-No HT.

The rate performance of MoS_2_/CC-HT, MoS_2_/CC-No HT, and the carbon cloth are demonstrated in Figure 9a. By removing the capacity of the carbon cloth, the rate performance of MoS_2_ in MoS_2_/CC-HT and MoS_2_ in MoS_2_/CC-No HT is presented in Figure 9b. MoS_2_ in MoS_2_/CC-HT delivers the capacities of 775, 802, 826, 790, and 663 mAh g^−1^ at current densities of 0.1, 0.2, 0.5, 1, and 2 A g^−1^, demonstrating its good rate performance. When the current density returns to 0.1 A g^−1^, it delivers a capacity of 991 mAh g^−1^, which is higher than its initial capacity at the current density of 0.1 A g^−1^. MoS_2_ in MoS_2_/CC-No HT delivers the capacities of 902, 652, 550, 453, and 311 mAh g^−1^ at the current densities of 0.1, 0.2, 0.5, 1, and 2 A g^−1^. When the current density returns to 0.1 A g^−1^, it delivers the capacity of 654 mAh g^−1^, which is lower than its initial capacity at the current density of 0.1 A g^−1^. The larger volume change of 1T phase MoS_2_ during charge–discharge leads to the worse capacity retention performance of MoS_2_/CC-No HT.

**Figure 10:**
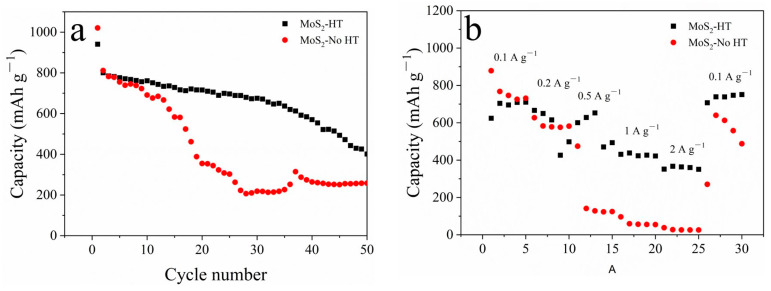
MoS_2_-HT and MoS_2_-No HT powder: (**a**) cyclic performance; (**b**) rate performance.

Figure 10 demonstrates the cyclic and rate performance of MoS_2_-HT and MoS_2_-No HT powders. From Figure 10a, it can be seen that MoS_2_-HT powder has a better capacity retention performance than MoS_2_-No HT powder. MoS_2_-HT delivers a capacity of 401 mAh g^−1^, while MoS_2_-No HT delivers a capacity of 258 mAh g^−1^ at a current density of 0.1 A g^−1^ after 50 cycles. During charge–discharge, 1T phase MoS_2_-No HT undergoes a larger volume change than 2H phase MoS_2_-HT, which leads to its worse capacity retention performance. From Figure 10b, at the gradually increasing current densities of 0.1, 0.2, 0.5, 1, and 2 A g^−1^, MoS_2_-HT delivers the capacities of 709, 616, 601, 427, and 363 mAh g^−1^, while MoS_2_-No HT delivers the capacities of 746, 578, 128, 57, and 27 mAh g^−1^.

**Figure 11:**
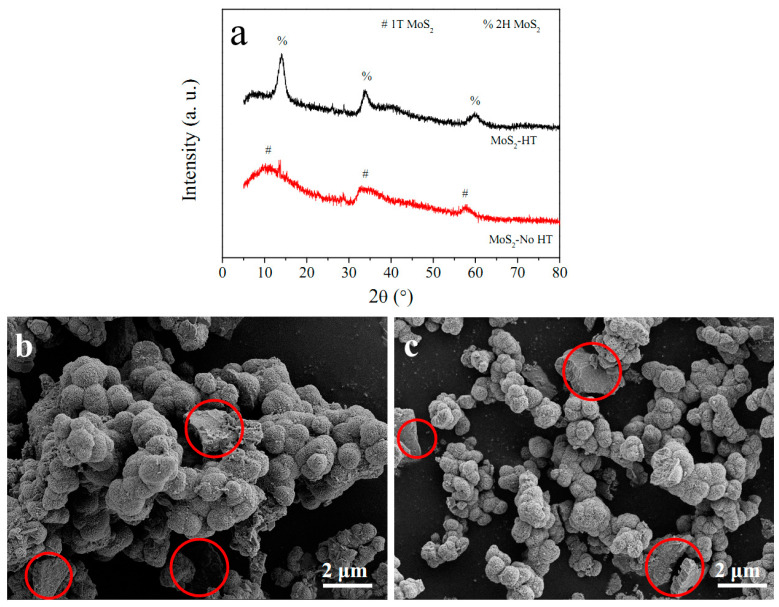
(**a**) XRD patterns of MoS_2_-HT and MoS_2_-No HT; SEM images of (**b**) MoS_2_-No HT and (**c**) MoS_2_-HT.

Figure 11a shows the XRD patterns of MoS_2_-HT and MoS_2_-No HT powders. It can be seen that MoS_2_-HT is 2H phase, while MoS_2_-No HT is 1T phase, indicating that the carbon cloth substrate will not change the phase of the loaded MoS_2_. Figure 11b,c demonstrate the SEM image of MoS_2_-No HT and MoS_2_-HT powder, respectively. Because heat treatment only changes the phase of MoS_2_, MoS_2_-No HT powder exhibits the same morphology as MoS_2_-HT powder. From Figure 11b,c, it can be seen that both MoS_2_-No HT powder and MoS_2_-HT powder consist of MoS_2_ flowers (area outside the red ring in Figure 11b,c) and MoS_2_ aggregates (area inside the red ring in Figure 11b,c). Because MoS_2_ in this paper is synthesized by a hydrothermal reaction in an aqueous solution of ammonium molybdate and thiourea, the strong polarity of water inevitably leads to the aggregation of the synthesized MoS_2_. Large-sized MoS_2_ aggregates will undergo fragmentation, pulverization, and detachment during the charge–discharge, resulting in their capacity fading. The carbon cloth substrate causes the loaded MoS_2_ to form a mutually separated nanosheet array. The non-agglomerated MoS_2_ nanosheet array will increase electrochemical reaction sites and promote the diffusion of lithium ions in the electrode. Thus, MoS_2_/CC-HT delivers higher capacity than MoS_2_-HT, while MoS_2_/CC-No HT delivers higher capacity than MoS_2_-No HT.

**Figure 12:**
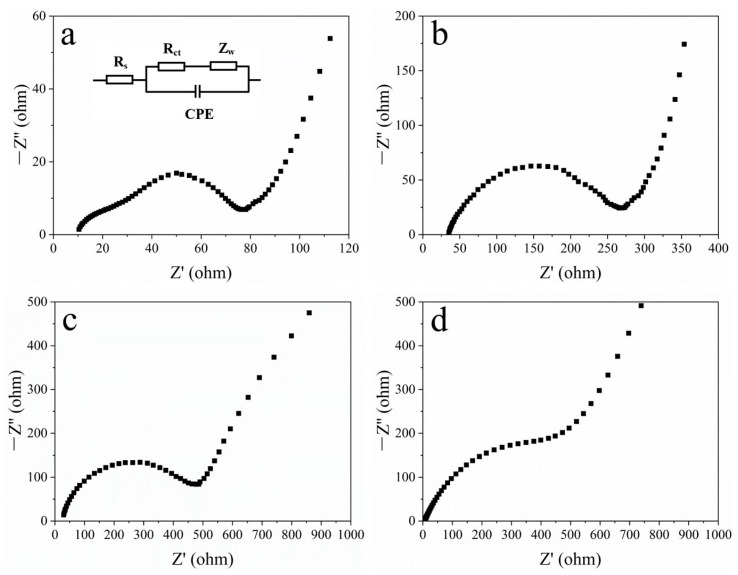
EIS patterns of: (**a**) MoS_2_/CC-HT (equivalent circuit in inset); (**b**) MoS_2_/CC-No HT; (**c**) MoS_2_-HT; (**d**) MoS_2_-No HT.

Figure 12 presents the EIS patterns of MoS_2_/CC-HT, MoS_2_/CC-No HT, MoS_2_-HT, and MoS_2_-No HT. In the mid-frequency region, the diameter of the semicircle in the Nyquist plot is related to the charge transfer resistance of the electrode [39,40]. The larger semicircle diameter means the larger charge transfer resistance of the electrode. The charge transfer resistances of MoS_2_/CC-HT, MoS_2_/CC-No HT, MoS_2_-HT, and MoS_2_-No HT are 60.2, 245.6, 466.8, and 575.5 Ω, respectively. The carbon cloth has good conductivity. The conductivity of the loaded MoS_2_ nanosheets can be enhanced by the carbon cloth substrate, thereby reducing their charge transfer resistance. In the low-frequency region, the slope of the oblique line in the Nyquist plot is related to the diffusion rate of lithium ions in the electrode. The steeper the slope of the oblique line, the faster the rate of diffusion of lithium ions into the electrode [41]. Apparently, the diffusion rate of lithium ions in MoS_2_/CC-HT is faster than that in MoS_2_-HT, and the diffusion rate of lithium ions in MoS_2_/CC-No HT is faster than that in MoS_2_-No HT. The mutually separated MoS_2_ nanosheets grown on the surface of the carbon cloth will promote the diffusion of lithium ions in the electrode.

**Figure 13:**
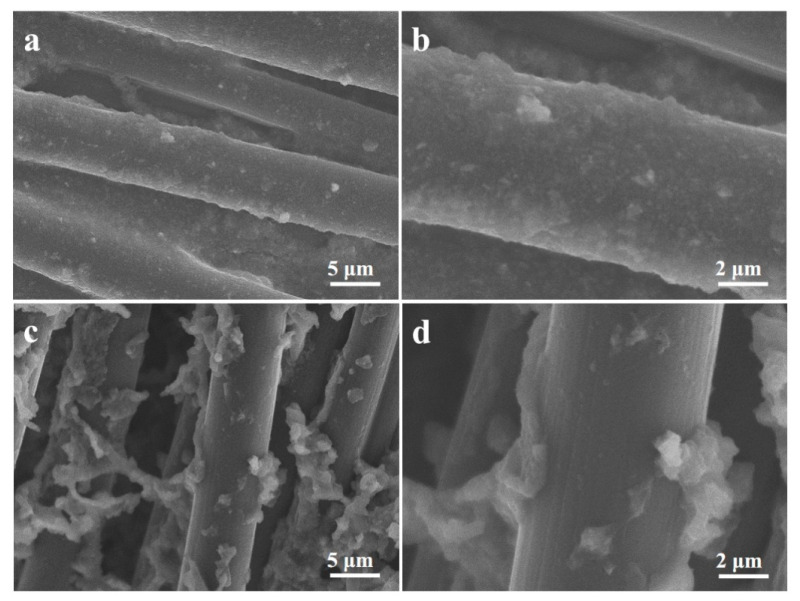
SEM images of: (**a**,**b**) MoS_2_/CC-HT after cycling; (**c**,**d**) MoS_2_/CC-No HT after cycling.

Figure 13a,b provide the SEM images of MoS_2_/CC-HT after 50 cycles at a current density of 0.1 A g^−1^. The MoS_2_ layer still firmly adhered to the surface of the carbon cloth without shedding. In contrast, for MoS_2_/CC-No HT after cycling, a large amount of detachment occurred in the MoS_2_ layer (Figure 13c,d). This is the reason why MoS_2_/CC-HT has a better capacity retention performance than MoS_2_/CC-No HT.

## 4. Conclusions

In this paper, ammonium molybdate and thiourea were employed as raw materials to synthesize a MoS_2_/carbon cloth composite via a hydrothermal reaction. Under the S/Mo molar ratio of 5, the grown MoS_2_ on the surface of the carbon cloth is ultra-thin nanosheets. MoS_2_ in MoS_2_/CC-No HT synthesized using a hydrothermal reaction is the 1T phase. After heat treatment, it transforms into the 2H phase while maintaining its morphology.

As an anode material for LIBs, the MoS_2_ in MoS_2_/CC-HT delivers a continuously increasing capacity of 1075 mAh g^−1^ at a current density of 0.1 A g^−1^ after 50 cycles, while the MoS_2_ in MoS_2_/CC-No HT delivers a fading capacity of 528 mAh g^−1^. With heat treatment, MoS_2_ on the surface of the carbon cloth is transformed from the 1T phase to the 2H phase with a better capacity retention performance, resulting in the better capacity retention performance of MoS_2_/CC-HT.

MoS_2_ powder synthesized without the carbon cloth substrate consists of MoS_2_ flowers and MoS_2_ aggregates. Aggregates reduce the electrochemical reaction sites and hinder the diffusion of lithium ions in the electrode, which decrease their electrochemical performance. The carbon cloth substrate can improve the conductivity of loaded MoS_2_ nanosheets and prevent them from agglomerating, which increases their capacity, reduces their charge transfer resistance, and promotes the diffusion of lithium ions in the electrode.

A carbon cloth with good conductivity and mechanical flexibility is an appropriate current collector for a flexible LIBs electrode. In this paper, a simple heat treatment route was employed to synthesize a MoS_2_/carbon cloth composite with a high capacity and good capacity retention performance. This composite is expected to be applied in wearable electronic devices.

## Data Availability

Data are contained within the article.

## Supplementary Materials

The following supporting information can be downloaded at: https://www.mdpi.com/article/10.3390/ma16247678/s1, Table S1: Centre, FWHM and percentage area (area%) for the deconvoluted peaks of Mo 3d. Table S2: Centre, FWHM and percentage area (area%) for the deconvoluted peaks of S 2p. Table S3: Centre, FWHM and percentage area (area%) for the deconvoluted peaks of C 1s.
