# Supplementary material for: The Effect of Heat Treatment after Hydrothermal Reaction on the Lithium Storage Performance of a MoS2/Carbon Cloth Composite
Source: Materials (Basel). 2023 Dec 17;16(24):7678. doi: 10.3390/ma16247678 (PMC10745091; doi:10.3390/ma16247678)
Supplement: Supplementary file 1 [file materials-16-07678-s001.zip › materials-2728733-supplementary.pdf]

Supporting Information

The Effect of Heat Treatment after Hydrothermal Reaction on the Lithium Storage Performance of a MoS_2/Carbon Cloth Composite

Xintong Li, Chonggui Li * and Qi Yang

School of Materials Science and Engineering, Shanghai University of Engineering Science, Shanghai 201620, China

* Correspondence: chongguili@sues.edu.cn

Table S1. Centre, FWHM and percentage area (area%) for the deconvoluted peaks of Mo 3d.

Peak & Identity	Centre (eV)	FWHM (eV)	Area %
S 2s	226.78	2.00	14.81
Mo $3d_{5/2}$ Mo^{4+}	229.61	1.24	47.23
Mo $3d_{3/2}$ Mo^{4+}	232.76	1.24	32.60
Mo $3d_{5/2}$ Mo^{6+}	230.98	1.26	3.17
Mo $3d_{3/2}$ Mo^{6+}	234.03	1.26	2.19

Table S2. Centre, FWHM and percentage area (area%) for the deconvoluted peaks of S 2p.

Peak & Identity	Centre (eV)	FWHM (eV)	Area %
S $2p_{3/2}$	162.40	1.11	66.19
S $2p_{1/2}$	163.59	1.11	33.81

Table S3. Centre, FWHM and percentage area (area%) for the deconvoluted peaks of C 1s.

Peak & Identity	Centre (eV)	FWHM (eV)	Area %
C-C	284.80	1.26	88.23
C-O	285.77	1.26	11.77
